# Characteristics and risk factors of pharmacist misconduct in New Zealand: a retrospective nationwide analysis

**DOI:** 10.1186/s12913-024-10591-2

**Published:** 2024-02-20

**Authors:** Yufeng Wang, Sanyogita (Sanya) Ram, Shane Scahill

**Affiliations:** School of Pharmacy, Faculty of Medical and Health Sciences, University of Auckland, Level 3, Building 503, 85 Park Road, Grafton, Auckland, 1023 New Zealand

**Keywords:** Misconduct, Pharmacist, Risk factors, Disciplinary matters

## Abstract

**Background:**

Professional misconduct has evolved into a worldwide concern, involving various forms and types of behaviours that contribute to unsafe practices. This study aimed to provide insights into the patterns characterising pharmacist misconduct and uncover underlying factors contributing to such instances in New Zealand.

**Methods:**

This research examined all cases of pharmacist misconduct sourced from the Health Practitioners Disciplinary Tribunal (HPDT) database in New Zealand since 2004. Characteristics of the sampled pharmacists and cases were extracted, followed by a systematic coding of the observed misconduct issues. Identification of risk factors was accomplished through content analysis techniques, enabling an assessment of their prevalence across various forms of misconduct.

**Results:**

The dataset of pharmacist misconduct cases comprised 58 disciplinary records involving 55 pharmacists. Seven types of misconduct were identified, with the most commonly observed being quality and safety issues related to drug, medication and care, as well as criminal conviction. A total of 13 risk factors were identified and systematically classified into three categories: (1) social, regulatory, and external environmental factors, (2) systematic, organisational, and practical considerations in the pharmacy, and (3) pharmacist individual factors. The most frequently mentioned and far-reaching factors include busyness, heavy workload or distraction; health impairment issues; and life stress or challenges.

**Conclusions:**

The patterns of pharmacist misconduct are complicated, multifaceted, and involve complex interactions among risk factors. Collaborative efforts involving individual pharmacists, professional bodies, responsible authorities, policy-makers, health funders and planners in key areas such as pharmacist workload and well-being are expected to mitigate the occurrence of misconduct. Future research should seek to uncover the origins, manifestations, and underlying relationships of various contributing factors through empirical research with appropriate individuals.

## Background

In the health and social care context, misconduct represents a violation of crucial organisational and professional norms, posing a significant threat to the well-being of organisations and the individuals who serve them [1]. The consequence of such misconduct lies in manipulating the trust placed in professionals by patients, subsequently exposing them to potential harm [2]. In the United Kingdom (UK), clinical negligence claims incur a substantial financial burden of £69.6 billion for the year 2022/2023 [3]. Furthermore, this detrimental behaviour casts a shadow over the reputations of regulatory bodies and fellow practitioners, gradually undermining public confidence in the healthcare sector and its establishments [1]. Therefore, it is imperative to comprehensively understand the various manifestations of misconduct and its underlying causes. Such insights hold value for regulators in devising effective regulatory measures and establishing long-term preventive tactics.

Major countries worldwide have proactively implemented regulatory frameworks to identify and penalise professional misconduct, resulting in a significant body of empirical research within the healthcare sector. Global studies have primarily focused on large-scale descriptive statistics, examining patterns of misconduct. These studies have recently investigated high-risk practitioner identification and predictive analysis [4–8]. Several factors have been identified as associated with misconduct, including a practitioner’s older age, male gender, longer in practice, claims history, and heavy workload [9–13]. However, existing research has mainly centred on medical physicians, with limited studies involving pharmacists. According to an Australian study conducted in 2020, pharmacists were overrepresented in disciplinary cases, appearing at nearly twice their expected proportion among practitioners [14], underscoring the need for further research on misconduct within the pharmacist profession.

In New Zealand, the Health Practitioners Competence Assurance Act 2003 (HPCAA) established a national scheme to regulate the registration, accreditation and discipline of health professionals [14]. The Act directs responsible authorities, including the Director of Proceedings (DP) and the Professional Conduct Committee (PCC), to bring disciplinary charges against health practitioners suspected of committing professional misconduct, which also applies to pharmacists [15]. These charges are brought before the Health Practitioners Disciplinary Tribunal (HPDT or Tribunal), the sole body responsible for hearing and determining disciplinary proceedings [16]. If a practitioner is found in violation of any of the seven categories outlined in HPCAA 2003 s.100 (as detailed in Table 1), the HPDT may impose one or more penalties to discipline the professional. Almost all HPDT decisions, with a few exceptions, are publicly accessible on its website [15]. These records encompass case summaries and decisions, offering extensive information on facts, charges, legal principles, opinions, and the penalties imposed by the HPDT. Therefore, the HPDT database offers a valuable window for gaining insights into instances of health practitioner misconduct.

**Table 1:** Grounds on which health practitioners may be disciplined according to HPCAA 2003

100 Grounds on which health practitioner may be disciplined
(1) The Tribunal may make any 1 or more of the orders authorised by Sect. 101 if, after conducting a hearing on a charge laid under Sect. 91 against a health practitioner, it makes 1 or more findings that—
(a) the practitioner has been guilty of professional misconduct because of any act or omission that, in the judgment of the Tribunal, amounts to malpractice or negligence in relation to the scope of practice in respect of which the practitioner was registered at the time that the conduct occurred; or (b) the practitioner has been guilty of professional misconduct because of any act or omission that, in the judgment of the Tribunal, has brought or was likely to bring discredit to the profession that the health practitioner practised at the time that the conduct occurred; or (c) the practitioner has been convicted of an offence that reflects adversely on his or her fitness to practise; or (d) the practitioner has practised his or her profession while not holding a current practising certificate; or (e) the practitioner has performed a health service that forms part of a scope of practice of the profession in respect of which he or she is or was registered without being permitted to perform that service by his or her scope of practice; or (f) the practitioner has failed to observe any conditions included in the practitioner’s scope of practice; or (g) the practitioner has breached an order of the Tribunal under Sect. 101.

This study aims to analyse HPDT disciplinary reports to develop a comprehensive understanding of the characteristics and risk factors associated with pharmacist misconduct in New Zealand. In this context, misconduct refers to actions that result in pharmacists being charged and facing disciplinary measures. The goal of this study is to enhance regulatory measures and educational strategies within the pharmacy profession.

## Methods

### Study design

This study used a mixed-methods approach to retrospectively analyse pharmacist misconduct records from the HPDT database. A combination of descriptive quantitative analysis and qualitative content analysis was performed to uncover the characteristics of pharmacist misconduct, identify the underlying contributing factors, and explore their interrelationships. Ethical approval was not required as all information used is publicly available.

### Data source

Data for this study were obtained from the publicly accessible HPDT website database (https://www.hpdt.org.nz/Search-Decisions?). The search was specifically restricted to the professional category of Pharmacists, covering the period from January 1, 2004, to December 31, 2021. A total of 58 records were identified. The data source included individual entries within the disciplinary records, case summaries, and narrative full decisions or substantive decisions.

### Data extraction

Each entry in the disciplinary records was considered as an individual unit and sequentially labelled from HPDT01 through to HPDT58, establishing a unique Case ID in chronological order. An Excel™ spreadsheet was utilised to extract the following sample information based on the individual entry and case summary: Case characteristics (case ID, file number, decision number, decision date, agency laying the charge, charge characteristics, charge details, charge grounds, penalties); Pharmacist characteristics (gender, practice scope, practice location).

### Data coding and analysis

We developed a preliminary taxonomy by consolidating categories from prior studies to code the misconduct issue [17, 18]. One author reviewed the charge characteristics and charge details outlined in each HPDT case summary, applying the initial typology to categorise misconduct in these cases, adjusting and modifying categories as necessary. After discussion and verification by the entire research group, we identified a final set of 29 misconduct issues, which were further grouped into seven categories: drugs/medication/care: quality and safety, criminal conviction, registration/certification, management/supervision, forgery/fraud/dishonesty, relationship/communication/rights, and other individual behaviour. The issues were not singular, there were multiple issues associated with each case.

Subsequently, all narrative full decisions or substantive decisions for each HPDT case were imported into QSR NVIVO™ 10 for qualitative coding of risk factors. The reports were consistently named to match the data extraction items, ensuring uniformity. In cases where different records referred to the same decision document, the report names were combined, for example, HPDT22&23.

A conventional content analysis technique was applied to investigate risk factors contributing to pharmacist misconduct [19]. The narrative reports were read multiple times to gain a general understanding of each case. All meaningful texts addressing the risk factors for pharmacist misconduct were condensed and labelled into codes. These codes were then grouped to form categories and themes, expressing underlying meanings within the text by comparing similarities and differences among the codes [20–22]. 

To better understand the risk factor patterns, we conducted a further cross-analysis of the risk factor codes and the original text, determining the misconduct issues influenced by these risk factors. The relationships were documented in an Excel™ spreadsheet. In cases where the description of a single risk factor corresponded to multiple misconduct issues, each individual relationship was recorded.

The rigour of data and findings was ensured by considering credibility, dependability, confirmability, and transferability [23]. The measures applied included a continuous 10-month engagement and ongoing observations, systematic compilation of coding notes, peer debriefing, and regular member checks with all emergent codes, findings and notes discussed, checked, and reviewed weekly during research team meetings, including a PhD student and two supervisors with expertise in pharmacy, organisational management, and law [23, 24]. 

## Results and key findings

### Sample characteristics

The database search yielded 58 disciplinary records involving 55 pharmacists. Among them, one pharmacist faced two disciplinary matters, and another was subject to three charges. Male pharmacists had over three times the rate of charges compared to female pharmacists. Additionally, two pharmacist interns were involved in misconduct cases (Table 2).

**Table 2:** Characteristics of the study sample (*n* = 58)

Sample Characteristics	N (%)
**Pharmacist Involved**	**55 (100)**
**Gender**	
Female	12 (21.82)
Male	45 (77.59)
Unknown	1 (1.72)
**Practice Scope**	
Pharmacist	56 (96.55)
Intern (trainee) Pharmacist	2 (3.45)
**Practice Location**	
Auckland	28 (48.28)
Dunedin	5 (8.62)
Christchurch	4 (6.90)
Palmerston North	3 (5.17)
Wellington	2 (3.45)
Others or Disclosure	16 (27.59)
**Cases**	**58 (100)**
**Agency Laying the Charge**	
DP	2 (3.45)
PCC	56 (96.55)
Grounds of Charge*	
S.100(1)(a) Professional misconduct malpractice or negligence	33 (56.90)
S.100(1)(b) Discredit	34 (58.62)
S.100(1)(c) Conviction adversely reflecting on fitness	18 (31.03)
S.100(1)(d) Practising without holding a current practising certificate	11(18.97)
Penalty*	
Costs	50 (86.21%)
Censure	47 (81.03%)
Conditions on practice	26 (44.83%)
Fine	21 (36.21%)
Suspension period	18 (31.03%)
Registration cancellation	13 (22.41%)

*Categories are not mutually exclusive

The majority of charges (96.55%) were laid by PCC. The most common grounds for professional misconduct were acts or omissions constituting malpractice or negligence within the practitioner’s scope of practice (S.100(1)(a)) and acts or omissions bringing discredit to the profession (S.100(1)(b)), both observed in over half of cases. Convictions of offences reflecting adversely on fitness to practice (S.100(1)(c)) and practising without a current practising certificate (S.100(1)(d)) were reported in 18 (31.03%) and 11(18.97%) cases, respectively.

The most frequently imposed penalties were costs (86.21%) and censure (81.03%). However, more severe penalties, such as suspension period (31.03%) and registration cancellation (22.41%), were comparatively less common.

### Misconduct issues

As presented in Table 3, the most common misconduct issues were related to the quality and safety issues related to drug/medication/care, accounting for nearly 40% of cases (*n* = 23, 39.66%). Following closely, criminal conviction constitutes the next most prevalent misconduct issue, reported in 18 cases (31.03%). Equally remarkable were misconduct issues involving management/supervision and practising without a current practising certificate, both representing approximately 20% of cases ((*n* = 12, 20.69% each). Another noteworthy concern was forgery/fraud/dishonesty (*n* = 11, 18.97%), followed by issues concerning relationships/communication/rights (*n* = 6, 10.34%). Additionally, 10 cases (17.24%) reported pharmacists’ other individual inappropriate behaviour.

**Table 3:** Main issues of misconduct

Main issues of misconduct	N	%
**Drug/Medication/Care: Quality and Safety**	**23**	**39.66%**
Inadequate/inappropriate dispensing	14	24.14%
Inappropriate administration and/or misuse of drugs	8	13.79%
Inadequate or failure to record	7	12.07%
Inappropriate storage of drugs	4	6.90%
Failure to confer with prescriber	3	5.17%
Inappropriate packaging/labelling of drugs	2	3.45%
Inadequate/inappropriate treatment care	1	1.72%
**Criminal conviction**	**18**	**31.03%**
Dishonest/forgery/inappropriate access with intent of pecuniary advantage	8	13.79%
Theft	4	6.90%
Inappropriate manufacturing/possession/administration of drugs	4	6.90%
Inappropriate possession of objectionable material	1	1.72%
Assault and/or inappropriate force	1	1.72%
**Registration/Certification**	**12**	**20.69%**
Practising without a current practising certificate	10	17.24%
Practising while suspended from practising	2	3.45%
**Management/Supervision**	**12**	**20.69%**
Breach of codified professional standards in management	10	17.24%
Inadequate/Inappropriate staff training/supervision	3	5.17%
Failure to provide adequate staff levels	1	1.72%
**Forgery/Fraud/Dishonesty**	**11**	**18.97%**
Inappropriate claiming	6	10.34%
Providing false information or misleading authorities	4	6.90%
Falsification or dishonest use of documents	2	3.45%
**Relationship/Communication/Rights**	**6**	**10.34%**
Breach of patient/public privacy	3	5.17%
Inadequate/inappropriate communication/informed consent/information with patients	2	3.45%
Disparaging/derogatory comments to colleagues	2	3.45%
**Other individual inappropriate behaviour**	**10**	**17.24%**
Inappropriate behaviour	3	5.17%
Failure to engage/comply with authority	2	3.45%
Inappropriate financial gain	2	3.45%
Alcohol	1	1.72%
Inappropriate possession/unlawful use of drugs	1	1.72%
Inappropriate access to records	1	1.72%

Among specific misconduct issues, the most frequently reported problem was inadequate/inappropriate dispensing (*n* = 14, 24.14%). Practising without a current practising certificate and breach of codified professional standards in management were each involved in 17.25% of the cases (*n* = 10). Other issues reported in over 10% of cases included inappropriate administration and/or misuse of drugs (*n* = 8, 13.79%), dishonest/forgery/inappropriate access with intent of pecuniary advantage (*n* = 8, 13.79%), inadequate or failure to record (*n* = 7, 12.07%), and inappropriate claiming (*n* = 6, 10.34%).

### Risk factors contributing to pharmacist misconduct

The content analysis revealed 13 risk factors contributing to pharmacist misconduct, grouped into three categories: macro, meso, and micro, including social, regulatory and external environmental factors; systematic, organisational and practical factors in the pharmacy; and pharmacist individual factors. The distributions of risk factors, explanatory description and excerpt examples are presented in Table 4.

**Table 4:** Risk factors identified through the content analysis

Risk factors (No. of cases, %)	Explanatory description	Excerpt examples from HPDT reports*
**Social, regulatory and external environmental factors **(***N* = 6, 10.3%)**		
Policy with potential financial incentives (2, 3.45%)	The dispensing fee policies incentivised improper claims and the pursuit of unwarranted financial gains, particularly noticeable among profit-driven pharmacists.	“It was the PCC’s contention that the close control regime potentially provided a significant financial incentive for a pharmacist to dispense on a close control basis.” *(HPDT26&27)*
Media pressure (1, 1.72%)	As a result of a prior error at the pharmacy, this situation escalated the pharmacist’s stress and subsequently led to dispensing errors.	“It was stated that the pharmacy had been under intense media pressure due to prior errors, (these occurring before [the practitioner] was employed by the pharmacy)…It was acknowledged that the pharmacy in this case had been the subject of media attention.” (*HPDT03*)
Insufficient certification system (1, 1.72%)	The return-to-practice application system lacked a provision for the practitioner to accurately indicate the date of her practice resumption, which ultimately led to the engagement in forgery misconduct.	“A complicating factor in this case is that although [the practitioner] went online to complete the application for her APC, that application did not specifically ask her to declare that she was currently practising the profession as required under s 26(2)(a) of the Act. The “Return to Practice” form does not strictly meet these requirements. Although these provisions do not excuse [the practitioner] from her actions of submitting inaccurate information on the “Return to Practice” form, they do in part demonstrate the difficulty she had in reflecting her circumstances at that time when completing the form online.” *(HPDT55)*
Low socio-economic environment (1, 1.72%)	The challenging socio-economic context led the pharmacist to rely on a complex and unreliable computer system, resulting in a criminal conviction for document misuse aimed at financial gain due to dealing with disorganised and non-compliant patients.	“[The practitioner] described his pharmacy as being in a low socio-economic area with a high percentage of turnover coming from prescriptions and many clients with serious medical problems on Community Service Cards. Many of them were highly disorganised and non-compliant with taking their medicines.” [The practitioner] described the fact that there had been difficulties where, “…patients who were unable to receive dispensing of repeats because they were a day or so over the time limits for the funded dispensing of those repeats became irate and threatening.”…He therefore had his computer system generate a report of the repeat medication prescriptions which were close to expiry date…This system became more sophisticated with time with the medicines not being dispensed in later months but simply labels prepared. Towards the end of the period when [the practitioner] was running the system the prescriptions were simply put through the computer system and claimed from the government without either the labels or prescriptions being made up. If the patient came in to the pharmacy then the medicines were dispensed as required. (*HPDT04*)
Insufficient warning from authority (1, 1.72%)	The absence of warnings from regulatory bodies regarding past investigations led to a lack of awareness among pharmacists regarding the seriousness of the issue, contributing to misconduct in medication provision.	“[The practitioner] said that he believed that because Medsafe, HealthPac, and the Medical Association representatives had and were continuing to investigate [the doctor], he thought that those “higher authorities” had whatever situation they were investigating under control. As no instruction to discontinue dispensing had been issued, that suggested to him that everything regarding [the doctor]’s prescribing of Sudomyl had been investigated and no other steps were required.” *(HPDT09)*
**Systematic, organisational and practical factors in the pharmacy **(*N*** = 28, 48.28%)**		
Busyness, heavy workload or distraction (15, 25.96%)	In a busy and distracting practical environment, practitioners found it challenging to maintain proper standards, leading to reduced attention to detail, increased stress, depression, and drug use, ultimately heightening the risk of errors.	“There were times when [the practitioner] worked at the Pharmacy for 13.5 hours. Although the Tribunal is not asked to make any comment on the sensibility of this, it would certainly have meant that he would be tired from time to time and risks of mistakes increase.” *(HPDT47)* “[The practitioner]’s explanation for not starting an incident report, that he was “distracted” (by high volume checking and dispensing of prescriptions and medico-packs)…” *(HPDT39)*
Illegal, unethical or irresponsible employer (5, 8.62%)	Practitioners sometimes engage in wrongful behaviour by following illegal or unethical orders from their employers. Additionally, an irresponsible employer failing to ensure the practitioner had a current APC also contributed to misconduct.	“It is not for the Tribunal to make any finding against the employer; but the Tribunal notes that it is disappointing that the employer did not apparently take any responsibility to ensure that [the practitioner], while in that employment, had a current APC.” *(HPDT34)* “This evidence related of course to the evidence that the owner of Birkenhead Avenue Pharmacy, [the owner], was apparently acting in a way which raised such concerns, and the Practitioner’s position in the correspondence that it was [the owner]’s practices that he was implementing.” *(HPDT43)*
Inadequate pharmacy system (3, 5.17%)	Inadequate maintenance of repeat prescription records, absence of patient scripts, errors in data entry, and breakdowns in communication directly impeded the efficiency and accuracy of pharmacist practice.	“It would appear that the system which was in place at the relevant time was not programmed adequately to pick up such discrepancies. Such systems should be in place as referred to in [the pharmacist advisor]’s evidence (above) would be further enhanced by being able to check with the patient directly.” *(HPDT09)*
Unfavourable workplace culture (3, 5.17%)	Strained work culture and incidents of bullying induced pressure on the practitioner, undermining their confidence in professionalism, causing health deterioration and professional impairment.	“[The Practitioner] explained that, in addition to this pressure, he had lost confidence in his professionalism and, on reflection, acknowledges that he was in poor health. He stated that the nature of the working situation was, for him, basically a ‘toxic’ one.” (HPDT10)
Previous errors in the pharmacy (1, 1.72%)	As the root cause of the media pressure, prior pharmacy errors indirectly played a role in fostering pharmacist misconduct, as depicted in the aforementioned example.	“It was submitted for [the practitioner] that the background to the error was relevant. It was stated that the pharmacy had been under intense media pressure due to prior errors, (these occurring before [the practitioner] was employed by the pharmacy).” *(HPDT03)*
**Pharmacist individual factors **(*N*** = 16, 27.6%)**		
Health impairment (9, 15.52%)	Health impairment, including physical illness, mental health challenges, and substance abuse, often originates from personal life stressors or suboptimal work environments, indirectly contributing to pharmacist misconduct behaviours.	“[The Practitioner] reported to [The Psychiatrist] that she felt very stressed and low in mood and had become suicidal, therefore she wrote a number of prescriptions with a plan to overdose. [The psychiatrist]’ opinion was that [The Practitioner]’s ‘actions in writing the prescriptions were directly linked with her mental illness’.” *(HPDT44)* “[The Practitioner] had been ‘under considerable stress and in that state of anxiety, completed the online form for registration incorrectly.” *(HPDT55)*
Life stress or challenge (7, 12.07%)	Life stressors and challenges primarily act as predisposing factors for pharmacist health issues, indirectly influencing pharmacist misconduct behaviours.	“[The Practitioner] said his position was not helped by the fact that he does not have any family or other support network in New Zealand, and he described this as having resulted in a vicious cycle of depression and drug use.” *(HPDT31)* “My fault may be my empathetic nature to my family, friends, staff and customers. I was completely consumed by my friend’s impending death that I overlooked the requirement to have my application to the Pharmacy Council…” *(HPDT37)*
Opposition to authority or rule (2, 3.45%)	Pharmacists opposed to the Pharmaceutical Society or the Standard Operating Procedures (SOPs) resulted in their failure to adhere to established ethics and professional standards.	“[The Practitioner] reiterated that SOPs were not appropriate and gave examples of where he thought his own methods would suffice. He said, for example that he would visually inspect starting materials…” *(HPDT18)* “A key element of the Practitioner’s evidence involved explaining his long-standing differences with the Pharmaceutical Society of New Zealand. The reasons for these differences are unimportant. The Tribunal accepts that the Practitioner had a rooted objection to becoming a member of the Pharmaceutical Society. In his evidence, the Practitioner described himself as a “conscientious objector”. There is room for different views as to whether that description of his position is apt. “ *(HPDT36)*

*Examples were extracted from reports reviewed during the content analysis process. Identification of patients, practitioners or other stakeholders’ names was anonymised using square brackets

### Social, regulatory and external environmental factors

At the macro level, the first category of risk factors pertains to social, regulatory and external environmental factors, involving six cases (10.3%). Policy and regulatory factors feature prominently in several instances. In two disciplinary records (3.45%), PCC suggested that government subsidy policies regarding dispensing fees might have driven pharmacist misconduct toward inappropriate claiming and financial gain. Additionally, a specific case highlighted (1, 1.72%) how an insufficient certification system can be linked to instances of forgery misconduct among pharmacists, stemming from their inability to select the correct practice end-date when completing the return-to-practice application. In another case (1, 1.72%), authorities’ omission of warnings on prior investigations caused the pharmacist to underestimate the severity of the issue, influencing his decision-making and ultimately resulting in medication issues.

Expanding the scope to a broader context, socio-economic environmental factors (1, 1.72%) shed light on an underlying issue concerning disorganised and non-compliant patients, thereby disrupting the practice of pharmacists. In a separate case (1, 1.72%), a pharmacist practitioner emphasised that media pressure resulting from past pharmacy errors influenced his operations, consequently leading to dispensing errors.

### Systematic, organisational and practical factors in the pharmacy

The most common factors were situated within the systematic, organisational and practical factors in the pharmacy, accounting for nearly half of the 58 cases (28, 48.28%). Foremost among these was the issue of busyness, heavy workload or distraction (15, 25.96%). Many practitioners expressed that a bustling or distracting work environment made it challenging to maintain accurate records, increased the likelihood of errors, and even initiated a detrimental cycle of depression and drug use. For pharmacists in supervisory roles, the consequences of a busy practice were more severe, as it might involve the entire system they oversee not being operated properly (HPDT26&27).

Other noteworthy aspects involve employer (5, 8.62%) and cultural (3, 5.17%) influences. While there was no excuse for misconduct, some practitioners were reported to engage in wrongful behaviour due to compliance with their employers’ illegal or unethical instructions. Moreover, in a case where an irresponsible employer failed to ensure that the practitioner had a current APC, it served as a contributing factor to the pharmacist’s subsequent misconduct. In the pharmacy organisation, an unfavourable work culture not only gave rise to negative emotions and stress but, more significantly, triggered health problems among pharmacists.

In contrast, hardware-related issues were not prominently highlighted in this study. Only three cases identified inadequacies in the system design (3, 5.17%). Additionally, as a trigger of media pressure, previous pharmacy errors (1, 1.72%) indirectly contributed to pharmacist misconduct, as illustrated in the example provided above.

### Pharmacist individual factors

Pharmacist individual factors were reported in 16 cases, constituting over a quarter (27.6%) of the cases. A substantial portion of these factors were attributed to health impairments (9, 15.52%), where pharmacist practitioners stated that their physical illness, mental health issues, and drug abuse affected their professionalism and impeded their ability to practice competently. Life stress or challenge (7, 12.07%) primarily manifested as precursors to health impairment issues, often driven by factors such as family tensions, marital or workplace relationships, and unfavourable financial conditions.

Interestingly, two cases (3.45%) highlighted pharmacists’ opposition to authority or established rules, wherein these practitioners questioned the appropriateness of the Standard Operating Procedures (SOPs) in the pharmacy and advocated for their own methods. In another case, a practitioner described himself as a “conscientious objector” to the Pharmaceutical Society, resulting in a failure to update his certification within the timeframe.

### Risk factors across misconduct issues

**Fig. 1:**
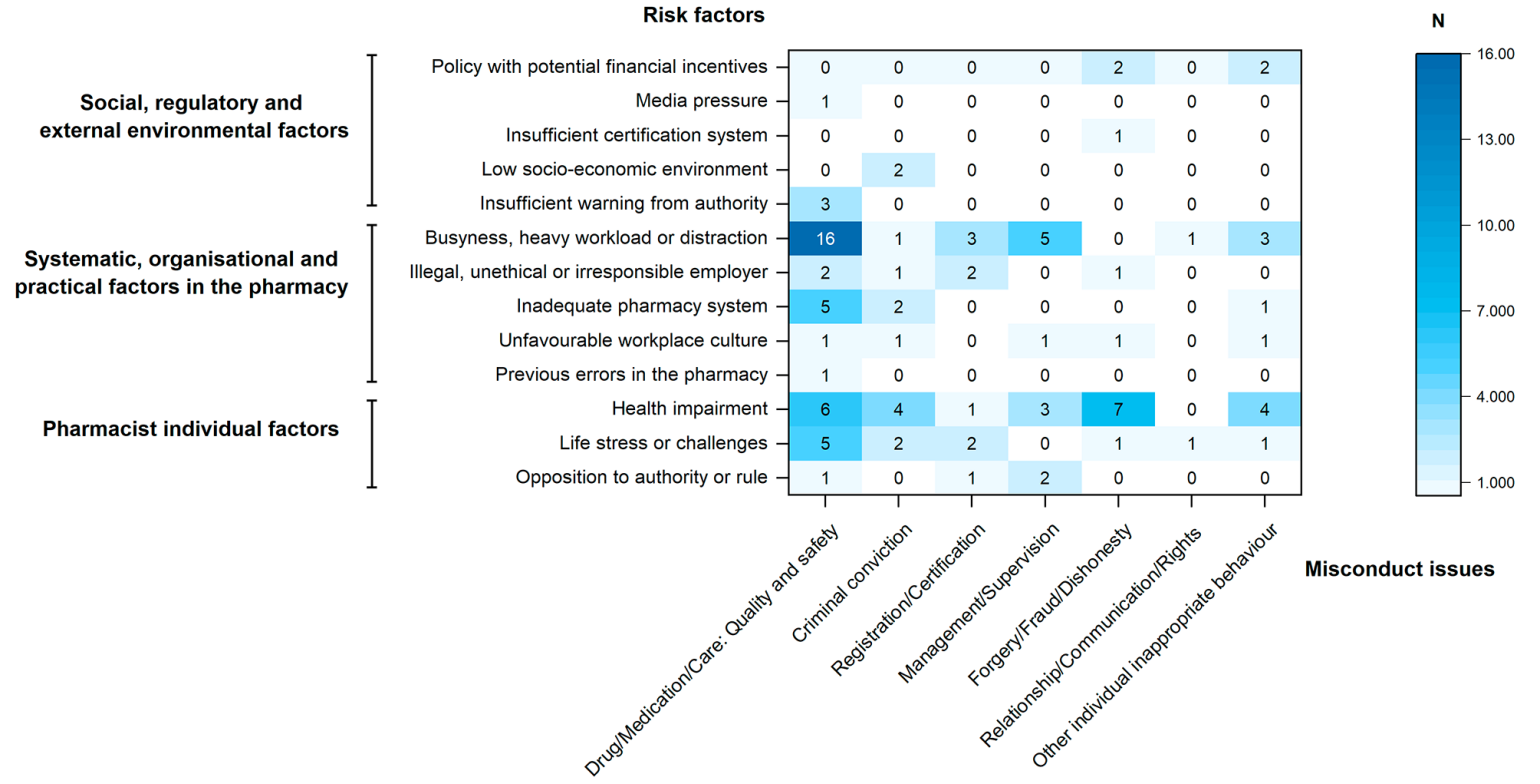
Relationships between risk factors and misconduct issues

As depicted in Fig. 1, a total of 97 relationships were identified between risk factors and misconduct issues. Overall, social, regulatory and external environmental factors had a relatively limited and specific impact on misconduct issues, affecting a narrower range. Conversely, systematic, organisational and practical factors in the pharmacy, and pharmacist-related individual factors exhibited a broader scope of influence, impacting a variety of misconduct types.

The most frequently mentioned and influential factor was busyness, heavy workload or distraction. This factor affected all types of misconduct except forgery/fraud/dishonesty issues, with a notable emphasis on quality and safety issues of drug/medication/care. Another equally outstanding factor was health impairment issues, primarily impacting cases involving forgery/fraud/dishonesty and drug/medication/care misconduct. It also pronounced impacts criminal conviction, other individual behaviour, and management/supervision issues. In addition to these two primary risk factors, quality and safety issues related to drug/medication/care also had considerable impact due to inadequate pharmacy systems and life stressors or challenges.

## Discussion

Analysing 58 cases of pharmacist misconduct brought before the HPDT, this study provides valuable insights into the characteristics and patterns of issues related to pharmacist misconduct. Aligning with prior studies conducted in Australia and the UK [25, 26], these findings indicate that pharmacist misconduct predominantly involves quality and safety issues related to drug, medication and care. The second most prevalent issue is conviction for criminal activity. A previous investigation in New Zealand revealed that compared to medical practitioners or nurses, a notably higher proportion of disciplinary actions among pharmacists arose from criminal activity [27]. Unexpectedly, over one-fifth of cases involve breaches of registration or certification, revealing a degree of oversight among pharmacists to proactively renew their professional certifications. In contrast, cases related to issues concerning relationship, communication, and rights appear to be less common. This indicates that most of these issues may not escalate to the point necessitating disciplinary action.

The findings of this research uncover three groups of underlying factors contributing to pharmacist misconduct: social, regulatory and external environmental factors; systematic, organisational and practical factors in the pharmacy; and pharmacist individual factors. Compared with previous studies where concerns related to supervision and organisational culture were infrequently identified [28], this study reverses this pattern. This shift could be linked to the sources of the research materials and the methodology undertaken. The utilisation of qualitative content analysis allows us to delve deeply into the systemic origins and provide valuable insights into qualitative factors within the organisation and system.

The impact of social, regulatory, and external environmental factors seems to be specific, influencing particular types of misconduct. For example, a policy offering potential financial incentives led pharmacists to engage in misappropriated financial gain through improper claiming. Similarly, an inadequate certification system resulted in pharmacists being unable to provide accurate information, thereby leading to misconduct issues of dishonesty related to providing false or misleading information to authorities. This implies that the influence of macro-level social, regulatory, or environmental factors is relatively narrow in scope, and regulatory bodies can effectively mitigate these effects by implementing timely measures or alternatives.

On the other hand, the findings reveal that the impact of pharmacy system and organisational factors, as well as pharmacist’s personal factors, is more intricate and extends to a broader spectrum of misconduct scope. Notably, busyness, heavy workload or distraction stand out as the most frequently cited risk factors. These elements have a considerable impact, affecting nearly all types of misconduct, especially those quality and safety issues related to drug, medication or care provision. A reasonable explanation is that every patient interaction presents a potential for adverse events to transpire, and it’s logical to anticipate a higher risk of such events in more intricate clinical encounters [13]. Saqib et al. suggest that practitioners burdened with excessive workloads often struggle to allocate sufficient time for their work, thereby adversely affecting the quality [29]. This can potentially lead to various misconduct issues, including management and communication problems.

Another notable risk factor pertains to health impairment. This research highlights health impairment issues play a prominent role in misconduct related to forgery/fraud/dishonesty. This finding aligns with those from a UK study that investigated dishonest behaviour among nurses and midwives [30], whereby dishonest behaviour including theft appeared to manifest as a consequence of their struggles to manage current fiscal pressures [30]. This study also reveals that health impairment impacts drug, medication and care provision quality and safety. Poor mental health states, including stress, depression, burnout, and poor overall well-being, have been associated with medical error [31, 32]. A recent study highlights that acute and recent injury may contribute to this risk, which mirrors these research findings and reinforces prior recommendations to intensify preventive efforts aimed at improving doctors’ health and well-being [33].

Despite evidence suggesting that pharmacists might exhibit inherently higher rates of substance use/abuse than other health professionals [34], the findings of this study fail to support this. Only a single case highlights issues related to inappropriate drug use. One plausible explanation could be that, while issues like mental distress and depression can go unnoticed by pharmacists in such psychological circumstances, pharmacists are medicine experts, and drug abuse presents a more conspicuous indicator. It is therefore more likely to attract the attention of pharmacists themselves. The heightened severity of drug abuse may lead to reporting and early intervention by pharmacists, potentially preventing the escalation to the level of professional misconduct brought before HPDT.

It is noteworthy that the impact of these risk factors is not a straightforward linear relationship; there are complex interrelationships among various factors. This is particularly evident with health impairment issues, where they not only serve as a risk factor for misconduct but also manifest as a consequence of other factors, including illegal, unethical or irresponsible employer, strained workplace culture, and life stresses or challenges such as financial difficulties and marriage/partner breakups. The multifactorial and interdependent relationships between factors have been supported by the literature [7, 35]. Future studies might benefit from utilising intersectionality analysis to determine whether the identified factors are independent or interconnected [10].

### Implications for regulation and practice

The findings of this study carry significant implications for shifting the focus towards organisational and systemic factors when attempting to address and alleviate misconduct issues by pharmacists. Identifying such factors is crucial for effectively allocating responsibility and reducing risk across the system. It helps to prevent the undue burden of resolving these issues from falling solely on individuals or organisations, especially when there may be a lack of authority and resources to address them [28, 36]. Many of those factors may not be easily tracked within pharmacies or across the sector and may require referral to national professional or regulatory bodies and policy-makers [28].

While the factors influencing misconduct are complex, strategic allocation of resources in targeted areas should help mitigate these issues. There is a need to foster positive and conducive work environments for pharmacists. Ensuring a favourable working atmosphere and addressing adequate staffing levels are essential to alleviate the burden of excessive workload. Additionally, efforts should be made to prioritise the overall health and well-being of pharmacists. Promoting a healthy lifestyle and incorporating positive psychological and well-being interventions have been shown to improve practitioners’ subjective well-being, improve patients’ perceptions of empathy, and ultimately enhance clinical outcomes [37].

Furthermore, there is a need to raise awareness and educate pharmacists, encouraging them to self-report health impairment so that responsible authorities, professional leadership bodies and pharmacy educators can identify risks early and provide necessary intervention and support. However, the fear of regulatory processes has been reported to create a barrier to practitioners accessing healthcare, but also exacerbating their adverse health outcomes [38]. A “therapeutic jurisprudence” approach is recommended to be applied in the regulatory process to promote the trust that practitioners and the profession place in regulators and regulatory processes [39, 40]. 

### Strengths and limitations

An important strength of this study is the application of qualitative content analysis to identify significant risk factors. The literature has applied largely quantitative approaches to identify statistically significant factors such as age and gender, using numerical data [41]. This study addresses the limitations of quantitative approaches in understanding complex phenomena such as organisational culture and the work environment. The implications of this study’s findings are particularly relevant in addressing this gap. Additionally, by embracing qualitative content analysis, this study not only offers a deep and rich perspective but also sheds light on the complex interactions between the various risk factors. Another novel aspect is the exploration of distribution patterns among various risk factors and misconduct issues.

This study has several limitations. Given the qualitative approach, some subjectivity is expected during the identification of factors and their relationships and the interpretation of texts. Data analysis relied on content analysis of disciplinary reports from the HPDT, for which a legal analytical approach needs to be applied, focusing on factual aspects of behaviours. As a result, the understanding of this study is limited to observable actions and does not encompass the understanding of the practitioner’s mindset and other dimensions beyond legal analysis. Furthermore, the limited sample size and reliance on a specific data source likely result in an incomplete representation and depth when attempting to understand the relationship between the risk factors and misconduct issues. To address this, future research must consider incorporating interviews or surveys to uncover a more comprehensive across-sector understanding.

## Conclusions

The present study uses pharmacist disciplinary cases to investigate critical areas of concern related to pharmacist misconduct risks. The study identifies multifaceted risk factors contributing to pharmacist misconduct, involving complex interactions. Addressing misconduct requires collaborative efforts from pharmacists, national pharmacy professional bodies, responsible authorities (regulators), policy-makers, health funders, and planners. By targeting resources in critical areas such as fostering a positive work environment and emphasising pharmacists’ overall health and well-being, the pharmacy sector can potentially reduce the occurrence of misconduct. Future studies should move from document analysis to data collection via interviews or surveys. This will enhance understanding of critical misconduct issues’ origins, manifestations, and interconnections, thereby contributing to developing highly effective intervention strategies and preventive measures, driving practical enhancements in pharmacy operations, and fostering patient safety.

## Data Availability

No datasets were generated or analysed during the current study.

## Abbreviations

HPDT: Health Practitioners Disciplinary Tribunal
HPCAA: Health Practitioners Competence Assurance Act 2003
DP: Director of Proceedings
PCC: Professional Conduct Committee
